# Vitamin D receptor polymorphisms and bone health after kidney transplantation

**DOI:** 10.3906/sag-1911-156

**Published:** 2021-04-30

**Authors:** Berfu KORUCU, Ajlan TÜKÜN, Özant HELVACI, Hasan YETER, Sevim GÖNEN, Galip GÜZ, Turgay ARINSOY

**Affiliations:** 1 Department of Nephrology, Faculty of Medicine, Gazi University, Ankara Turkey; 2 Department of Medical Genetics, Düzen Laboratories Group, İstanbul Turkey; 3 HLA Tissue Typing Laboratory, Faculty of Medicine, Gazi University, Ankara Turkey

**Keywords:** Kidney transplantation, *VDR* polymorphisms, osteoporosis, avascular necrosis, persistent hyperparathyroidism

## Abstract

**Background/aim:**

Bone disease is one of the most prominent complications after kidney transplantation. Bone diseases include osteoporosis, persistent secondary hyperparathyroidism, and avascular necrosis (AVN). We investigated the relationship between the polymorphisms of the vitamin D receptor ( *VDR* ) gene and bone diseases occurring after kidney transplantation.

**Materials and methods:**

The study consists of 234 kidney allograft recipients with a minimum follow-up of five years after kidney transplantation. Patients with glomerular filtration rates less than 30 mL/min/1.73m2, a history of parathyroidectomy, bisphosphonate use pre- or post-transplantation, and cinacalcet use posttransplantation excluded. We evaluated associations between the polymorphisms of the *VDR* gene (BsmI, TaqI, ApaI, FokI, and Cdx2), the first-year bone mineral density (BMD) scores, persistent secondary hyperparathyroidism, and AVN.

**Results:**

Patients with low BMD scores were significantly younger (P = 0.03) and had higher intact parathormone (iPTH) levels (P = 0.03). Cdx2 TT genotype significantly increases the risk of low BMD scores (OR: 3.34, P = 0.04). Higher phosphate levels were protective against abnormal BMD scores (OR: 0.53; P = 0.03). Patients with persistent hyperparathyroidism had significantly longer dialysis vintage and higher pretransplantation iPTH levels (P = 0.02 and P < 0.001, respectively). Cdx2, CT/TT, and ApaI CA/AA genotypes significantly increase the risk of persistent hyperparathyroidism (OR: 6.81, P < 0.001, OR: 23.32, P < 0.001, OR:4.01, P = 0.02, and OR: 6.30, P = 0.01; respectively). BsmI CT/TT genotypes were found to increase AVN risk with an HR of 3.48 (P = 0.03). Higher hemoglobin levels were also found to decrease AVN risk with an HR of 0.76 (P = 0.05).

**Conclusion:**

Certain *VDR* gene polymorphisms are associated with a higher risk for bone diseases after kidney transplantation.

## 1. Introduction

Kidney transplantation is the treatment of choice in end-stage kidney disease. Most of the chronic kidney disease (CKD) complications, such as anemia, cardiovascular mortality, and secondary hyperparathyroidism, tend to improve after kidney transplantation [1]. However, similar improvements are not observed in bone health [2]. Osteoporosis secondary to the use of glucocorticoids and other immunosuppressive drugs after solid organ or stem cell transplantation is firmly established. However, the osteoporosis mechanism after kidney transplantation is much more complicated. CKD – mineral and bone disorder (MBD) residues and persistent hyperparathyroidism considerably affect bone health [3,4]. Other suggested risk factors for osteoporosis after kidney transplantation are calcium and vitamin D deficiencies, age, female sex, and specific vitamin D receptor ( *VDR* ) polymorphisms (BsmI) [5,6].

The role in the skeletal metabolism of the steroid hormone Vitamin D’s nuclear receptor is well known. The vitamin D receptor (VDR) is a nuclear receptor that binds to DNA and acts as a transcription factor. The different receptor regions have distinctive functions, such as DNA binding, receptor dimerization, gene, and cofactor activation. The *VDR* gene consists of the non-coding 5′promoter region, exons 1A, 1B, 1C, exons 2 to 3 encoding the DNA-binding region, and exons 4 and 5 encoding the ligand-binding region. The most known *VDR* gene polymorphisms are BsmI (rs1544410), TaqI (rs731236), ApaI (rs7975232), FokI (rs2228570) and Cdx2 (rs11568820). These polymorphisms are associated with certain clinical outcomes due to their effects on the receptor structure or mRNA stability.

The most critical MBD improvement in kidney transplantation patients is the posttransplantation recovery of secondary hyperparathyroidism [7]. Secondary hyperparathyroidism does not improve in some patients. Thus, hypercalcemia, bone loss, and parathyroid hormone-suppressing treatment requirements continue in these patients. Calcitriol has a direct, suppressive effect on the parathyroid hormone by binding to the parathyroid gland’s VDR. CKD patients with *VDR* gene polymorphisms (BsmI) reported having relatively severe secondary hyperparathyroidism [8].

Avascular necrosis (AVN) is another disorder associated with CKD cases induced by glucocorticoid use with *VDR* gene BsmI polymorphisms due to more severe secondary hyperparathyroidism. AVN limits further use of glucocorticoid treatment and is a significant cause of disability. AVN occurs due to the temporary bleeding disorder of the bone and the reperfusion process. Angiogenesis is the first recovery phase after reperfusion. The second recovery phase is the cellular differentiation of mesenchymal cells to osteoblasts and osteoclasts. Inadequacy in the second phase causes significant morbidity. In the *VDR* knockout mice studies, VDRs demonstrated a direct cellular effect in osteoblast formation during bone regeneration [9]. Many studies investigate the mechanism of AVN susceptibility. However, AVN’s etiological, genetic, and pathogenic mechanisms have not been fully elucidated [10]. 

Vitamin D metabolism is crucial in bone diseases. This study investigates the possible associations between bone mineral density (BMD), persistent secondary hyperparathyroidism, AVN, and *VDR* gene polymorphisms (BsmI, TaqI, ApaI, FokI, and Cdx2) in kidney transplant recipients.

## 2. Material and methods

### 2.1. Study design and participants

The study was conducted between January 2018 and January 2019, with all living or cadaveric kidney allograft recipients giving informed consent. All recipients had five or more years of follow-up after their kidney transplantation in our institute. Patients were excluded who had an estimated glomerular filtration rate (eGFR) of less than 30 mL/min/1.73m2 as calculated by the Chronic Kidney Disease Epidemiology Collaboration formula. Other exclusion criteria were a history of parathyroidectomy, a history of malignancy, anorexia, malnutrition, and hypo- or hyperthyroidism.

Patients’ demographic features, primary renal disease, dialysis vintage, and comorbid conditions were evaluated. We recorded the dose and duration of the previous and current medical treatments. For each subject, we calculated the lifetime cumulative glucocorticoid dose that included the pretransplantation period.

### 2.2. Materials and measurements

The patients’ laboratory parameters of the pretransplantation period, first-year posttransplantation, and simultaneous with AVN, if applicable, were evaluated retrospectively. Dual-energy x-ray absorptiometry (DXA Horizon; Hologic Inc., Marlborough, MA, USA) was used with the unit of g/cm2 to examine the BMD of the posteroanterior position of the lumbar vertebrae L1-4, left proximal femoral neck, and total hip. The World Health Organization (WHO) classification for osteopenia and osteoporosis in terms of Z and T scores was used to determine bone health. For premenopausal women and men under the age of 50, the Z score was considered. For postmenopausal women and men over the age of 50, the T score was considered. Z or T score of ≤ –2.5 indicates osteoporosis. Z or T score between −2.5 and –1.0 indicates osteopenia. Z or T score of ≥ –1.0 indicates a normal bone mass [11]. For men under 50 years of age, a Z score < –2 is considered “below the expected range for age” according to the International Society for Clinical Densitometry (ISCD) recommendations [12]. The subjects’ BMD scores were divided into two groups: Normal BMD and abnormal BMD (osteopenia or osteoporosis) according to the femur neck or the lumbar total BMD scores. Patients with a femoral neck or lumbar total BMD scores < –1 were included in the abnormal BMD group. The persistent hyperparathyroidism group consisted of cases with serum iPTH levels > 65 pg/mL during their first-year posttransplant [13]. AVN cases were diagnosed using the x-ray examination, bone scintigraphy, or magnetic resonance imaging modalities.

### 2.3. Genotyping

We drew an extra 5 cc of blood from the participants’ antecubital vein during the sampling for one of their routine controls. We immediately transferred the draws to EDTA containing tubes. Since the study was carried out on the stable DNA molecule, blood samples were refrigerated at –80 °C and studied collectively at the end of the study.

DNA was extracted from whole blood using the Zinexts MagPurix Extraction Kit (ZP02001) (Zinexts Life Science, New Taipei City, Taiwan). A pair of polymerase chain reaction (PCR) primers were designed for each studied polymorphism. Template sequences are obtained from the Ensembl database Ensembl Project (2021). Ensembl Database [online]. Website http://www.ensembl.org/ [accessed date 00 Month Year]., and the transcript ID used for the *VDR* gene is ENST00000549336 (RefSeq ID: NM_000376). Primers are designed in-house by using Primer 2.0 software (Scientific and Educational Software).  The primers and the amplicon sizes obtained from the PCR are shown in Table 1. PCR amplification of the regions was performed using the designed primers. The PCR conditions are shown in Table 2. The reaction results were effectively visualized with a 2% agarose gel electrophoresis. For each sample, the PCR products were pooled by mixing them approximately equal with a consideration of the reaction yield. The PCR pools generated for each sample were purified according to the manufacturer’s recommended method (MN-NucleoFast 96, Macherey-Nagel, Düren, Germany). The measured and purified PCR pools (Nanodrop-ND1000, Thermo Fisher Scientific,Waltham, MA, USA) were set to 0.2 ng/µL in accordance with the sequencing kit used. The next-generation sequence was performed using the Miseq device of Illumina, California, USA. The samples were analyzed, and the genotypes were identified using the Miseq Reporter software from Illumina Inc. (San Diego, CA, USA) and the IGV 2.3 software developed by Broad Institute (California, USA).

**Table 1:** The primers and the amplicon sizes.

Primers	Sequence (5’->3’)	Amplicon size (bp)
Cdx 2 Forward	GGAATGAAAGAGGGAAGGAGGGAG	215
Cdx 2 Reverse	CTGTAGCAATGAAAGCAAACCAAGG	
FokI Forward	CACTGACTCTGGCTCTGACCGTG	177
FokI Reverse	CAGCCTTCACAGGTCATAGCATTG	
BsmI Forward	ACTGCCCTTAGCTCTGCCTTGC	259
BsmI Reverse	AAGGGTCACTGCACATTGCCTC	
ApaI and TaqI Forward	CAGGCAGTGGTATCACCGGTCAG	292
ApaI and TaqI Reverse	CGGCTAGCTTCTGGATCATCTTGG	

**Table 2:** The polymerase chain reaction conditions.

Content	Amount per Reaction (µL)
dH20	15
5x Tampon (Thermo Fisher Scientific Inc.,Waltham, MA, USA)	5
dNTP mixture, each 10 mM	0.5
Forward Primer (5 µM)	1
Reverse Primer (5 µM)	1
Phire II HS DNA Polymerase (Thermo Fisher Scientific Inc.)	0.5
Bar DNA (20–50 ng/µL)	2
Total	25

The study group’s genotypic distributions were analyzed according to their ages and their cumulative glucocorticoid doses. Considering the BMD scoring, we set the age cut-off to 50 years according to the WHO and ISCD recommendations. We set the cut-off value of the cumulative glucocorticoid dose to the study population’s mean dose. Compound heterozygosities were expressed as the percentage of the coexistence of the heterozygous or homozygous mutant type of other polymorphisms in heterozygous cases for each polymorphism.

### 2.4. Statistical methods

The numeric values are shown with the mean, standard deviation, medians with ranges, categorical data, frequencies, and percentages. We used the unpaired Student’s t-test to compare two groups for the data normally disturbed and independent samples t-test to compare more than two groups, and for the *VDR* gene polymorphism distributions’ frequency comparisons. We compared the categorical variables with chi-square test or Fisher’s exact test where applicable. We performed the Mann–Whitney U Test for the nonnormally distributed data. For the multivariate analysis, the possible factors identified with the univariate analyses were further entered into the logistic and Cox regression analyses. These factors were entered with the backward selection to determine the independent patient outcome predictors. The logistic regression was used for the first-year posttransplantation BMD scores and hyperparathyroidism. The Cox regression was used for AVN. A P value of less than 0.05 was considered statistically significant. We performed statistical analyses using the Statistical Package for Social Science (SPSS) (IBM Corp., Armonk, NY, USA) for personal computers version 21.0. 

## 3. Results

### 3.1. Power analysis

Power analysis was performed using the G*Power Statistical Software according to the wild (CC) and mutant (CT & TT) types of the *VDR* gene CDX2 polymorphism. The calculated power (1 – β) is 0.953. This value is calculated using the type I error (α) of 0.05, the total sample size of 234, the effect size of 0.48, and the two-sided alternative hypothesis (H1).

### 3.2. Osteopenia and osteoporosis

The baseline characteristics and *VDR* genotype distribution of patients are presented in Table 3. Patients with low BMD scores were significantly younger (P = 0.03). Cyclosporin-A (CsA) use was significantly frequent in the abnormal BMD group (P = 0.04). The patients with low first-year posttransplantation BMD scores had significantly higher simultaneous intact Parathormone (iPTH) levels (P = 0.03). The distribution of sex and primary diseases; the frequency of diabetics and mammalian target of rapamycin inhibitor (mTORi) users; dialysis vintage, cumulative doses of steroids, and laboratory parameters other than posttransplantation first-year iPTH levels were all similar between the study groups. 

**Table 3:** Baseline characteristics of patients grouped according to posttransplant first year BMD scores.

	Normal BMD(n = 153)	Abnormal BMD(n = 81)	P value
Age mean (±SD)	42.1 (±12.9)	38.5 (±12.2)	0.03
Sex, F/M (n)	57/96	31/50	0.07
Disease (n)	Unknown: 69 Gn/Vasc: 26 DM: 17 Others: 41	Unknown: 34 Gn/Vasc: 18 DM: 8 Others: 21	0.12
DM, n (%)	19 (12.4%)	6 (7.4%)	0.09
Vintage, (month) med (min-max)	19 (0–242)	22 (0–228)	0.90
CNI, n (%)	Tac: 131 (85.6%) CsA: 18 (11.7%)	Tac: 52 (64.1%) CsA: 13 (16.0%)	0.04
mTORi, n (%)	Eve: 9 (5.8%) Sir: 10 (6.5%)	Eve: 4 (4.9%) Sir: 6 (7.4%)	0.31
Steroid, mg mean (±SD)	3525.7 (±1050.3)	3460.9 (±808.3)	0.94
eGFR mean (±SD)	74.3 (±25.0)	75.6 (±24.5)	0.12
Ca, mmol/L mean (±SD)	2.38 (±0.15)	2.35 (±0.13)	0.68
P, mmol/L mean (±SD)	1.03 (±0.19)	1.00 (±0.16)	0.08
Mg, mmol/L mean (±SD)	0.85 (±0.10)	0.90 (±0.15)	0.60
25-OH-D, nmol/L mean (±SD)	80.6 (±28.2)	81.1 (±29.4)	0.65
Pre Tx iPTH, ng/L med (min-max)	600.2 (60.4–2853.2)	612.0 (128.1–1981.8)	0.95
1st year iPTH, ng/L med (min-max)	64.3 (14.5–2249.7)	71.4 (26.9–950.9)	0.03
LDL, mmol/L mean (±SD)	2.72 (±0.85)	2.68 (±0.93)	0.22
TG, mmol/L mean (±SD)	1.64 (±0.87)	1.67 (±0.94)	0.43
Cdx2 CC/CT/TT (%)	65.4/28.7/5.9	55.6/34.6/9.8	0.04
Fok1 AA/AG/GG (%)	7.2/43.1/49.7	12.3/42.0/45.7	0.31
Bsm1 CC/CT/TT (%)	40.5/47.7/11.8	28.4/34.6/37.0	0.04
Apa1 CC/CA/AA (%)	25.5/43.8/30.7	16.0/33.3/50.7	0.17
Taq1 AA/AG/GG (%)	43.8/45.7/10.5	30.8/34.6/34.6	0.10

The *VDR* gene’s Cdx2 TT genotype significantly increases the risk of low BMD scores with an odds ratio (OR) of 3.34 (min: 1.04 – max: 10.75; P = 0.04) when corrected for all possible risk factors. The higher serum phosphate levels were found to be protective against abnormal BMD scores (OR: 0.53, min: 0.30 – 0.95; P = 0.03), as demonstrated in Table 4.

**Table 4:** Logistic regression analysis of risk factors for low BMD scores.

		OR (95% CI)	P value
Cdx2 (ref: CC)	CT	1.77 (0.88-3.55)	0.10
	TT	3.34 (1.04–10.75)	0.04
FokI (ref: AA)	AG	0.90 (0.30–2.68)	0.85
	GG	0.72 (0.24–2.13)	0.55
BsmI (ref: CC)	CT	0.54 (0.09–3.03)	0.48
	TT	2.42 (0.12–45.70)	0.55
ApaI (ref: CC)	CA	1.69 (0.58–4.19)	0.33
	AA	1.99 (0.49–8.08)	0.33
TaqI (ref: AA)	AG	0.96 (0.19–4.76)	0.96
	GG	1.06 (0.07–15.67)	0.96
Age		0.97 (0.95–1.00)	0.93
Sex (Male)		1.03 (0.50–2.11)	0.93
Vintage (month)		1.02 (0.93–1.13)	0.57
Pre Tx iPTH (ng/L)		0.99 (0.99–1.00)	0.17
1st year iPTH (ng/L)		1.00 (0.99–1.00)	0.63
25-OH-D (nmol/L)		1.00 (0.97–1.03)	0.72
Ca (mmol/L)		0.64 (0.36–1.14)	0.13
P (mmol/L)		0.53 (0.30–0.95)	0.03
Mg (mmol/L)		0.43 (0.11–1.63)	0.21
eGFR		0.99 (0.98–1.01)	0.66
Steroid (mg)		1.00 (0.99–1.00)	0.49
Tac		0.64 (0.07–5.31)	0.68
CsA		0.41 (0.16–1.05)	0.06
Eve		1.32 (0.20–8.53)	0.76
Sir		0.78 (0.12–5.07)	0.80

### 3.3. Persistent hyperparathyroidism

The patients’ baseline characteristics and *VDR* genotype distribution are presented in Table 5. The patients with persistent hyperparathyroidism had a significantly longer dialysis vintage and high pretransplantation iPTH levels (P = 0.02 and P < 0.001, respectively). The calcium and alkaline phosphatase levels were significantly high in the persistent hyperparathyroidism group (P = 0.004 and P < 0.001, respectively) (Table 5). The distribution of age and sex were similar between groups. The levels of eGFR, phosphate, magnesium, and 25-OH-D vitamin were also similar between groups.

**Table 5:** Baseline characteristics of patients grouped according to the first-year posttransplant parathormone levels.

	Resolved hyperparathyroidism(n = 161)	Persistent hyperparathyroidism(n = 73)	P value
Age mean (±SD)	41.2 (±13.0)	40.6 (±12.2)	0.54
Sex F/M (n)	57/104	31/42	0.08
Vintage, (month) med (min-max)	42 (0–221)	52 (0–248)	0.02
eGFR mean (±SD)	74.5 (±24.3)	75.2 (±26.2)	0.34
Ca, mmol/L mean (±SD)	2.35 (±0.16)	2.49 (±0.17)	0.004
P, mmol/L mean (±SD)	1.03 (±0.16)	1.00 (±0.19)	0.72
Mg, mmol/L mean (±SD)	0.85 (±0.10)	0.85 (±0.11)	0.97
25-OH-D, nmol/L mean (±SD)	81.4 (±29.7)	79.4 (±25.9)	0.44
Pre Tx iPTH, ng/L med (min-max)	470.2 (60.2–2755.1)	745.1 (132.1–2340.0)	<0.001
ALP, µkat/L mean (±SD)	1.38 (±0.48)	1.63 (±0.93)	<0.001
Cdx2 CC/CT/TT (%)	74.5/23.0/2.5	34.3/47.9/17.8	<0.01
Fok1 AA/AG/GG (%)	8.1/47.2/44.7	11.0/32.9/56.1	0.21
Bsm1 CC/CT/TT (%)	36.6/44.8/18.6	35.6/39.7/24.7	0.56
Apa1 CC/CA/AA (%)	24.8/40.4/34.8	16.4/39.8/43.8	0.86
Taq1 AA/AG/GG (%)	41.0/42.2/16.8	35.6/41.1/23.3	0.95

The Cdx2 CT and TT genotypes of the *VDR* gene significantly increases the risk of persistent hyperparathyroidism [OR: 6.81 (min: 3.16 – max: 14.69), P < 0.001, and OR: 23.32 (min: 3.16 – max: 14.69), P < 0.001; respectively] when corrected for all possible risk factors. The CA and AA genotypes of ApaI also increase the risk of persistent hyperparathyroidism [O.R.: 4.01 (min: 1.24 – max: 112.95), P = 0.02, and O.R.: 6.30 (min: 1.45 – max: 27.42), P = 0.01; respectively] (Table 6). 

**Table 6:** Logistic regression analysis of risk factors for persistent hyperparathyroidism.

		OR (95% CI)	P value
Cdx2 (ref: CC)	CT	6.81 (3.16–14.69)	<0.001
	TT	23.32 (5.8–92.23)	<0.001
FokI (ref: AA)	AG	0.35 (0.11–1.12)	0.07
	GG	0.57 (0.18–1.77)	0.33
BsmI (ref: CC)	CT	0.10 (0.01–0.99)	0.06
	TT	0.10 (0.01–3.48)	0.20
ApaI (ref: CC)	CA	4.01 (1.24–12.95)	0.02
	AA	6.30 (1.45–27.42)	0.01
TaqI (ref: AA)	AG	4.12 (0.49–34.06)	0.18
	GG	4.02 (0.15–106.21)	0.40
Age		0.99 (0.96–1.02)	0.89
Vintage (month)		1.02 (0.93–1.13)	0.57
Pre Tx iPTH (ng/L)		1.07 (0.96–1.18)	0.18
25-OH-D (nmol/L)		0.99 (0.96–1.02)	0.51
Ca (mmol/L)		1.67 (0.90–3.08)	0.99
P (mmol/L)		0.79 (0.45–1.40)	0.43
Mg (mmol/L)		1.16 (0.30–4.51)	0.82
eGFR		0.98 (0.96–1.00)	0.11

### 3.4. Avascular necrosis

Table 7 demonstrates the patients’ baseline characteristics and *VDR* genotype distribution. Twenty-one subjects experienced AVN with a median of 85 months (min: 5 – max: 489). All patients had a minimum follow-up time of five years. Before the event, the patients with AVN had a cumulative steroid dose median of 5717.5 mg (min: 2752.5 – max: 56,490.0). The patients without AVN had a significantly higher cumulative steroid dose [12637.5 mg (min: 2605.0 – max: 79,250.5), P = 0.002] than the patients with AVN. Antiaggregant use was frequent in patients without AVN (P = 0.04). The patients’ hemoglobin levels were significantly lower in AVN patients than those without AVN (P = 0.03) (Table 7). The distribution of age, sex, primary diseases, and frequency of diabetics were similar between the groups. The CNI, mTORi, antilipidemic agent users, dialysis vintage, and laboratory parameters other than hemoglobin levels were also similar.

**Table 7:** Baseline characteristics of patients with or without AVN.

	No AVN (n = 213)	AVN (n = 21)	P value
Age mean (±SD)	41.0 (±12.9)	40.5 (±11.5)	0.68
Sex F/M (n)	81/132	7/14	0.33
Disease (n)	Unknown: 81 Gn/vasculitis: 38 Dm: 25Others: 69	Unknown: 11 Gn/vasculitis: 6 Dm: 0 Others: 4	0.24
DM, n (%)	25 (11.7%)	0 (0%)	0.09
Vintage, (month) med (min-max)	45.6 (0–238)	52.1 (0–231)	0.59
CNI, n (%)	Tac: 172 (80.7%) CsA: 22 (10.3%)	Tac: 14 (66.6%) CsA: 5 (23.8%)	0.09
mTORi, (n)	Eve: 13 (6.1%) Sir: 8 (3.7%)	Eve: 0 (0%) Sir: 2 (9.5%)	0.94
Steroid, mg mean (±SD)	12637.5 (2605.0–79250.5)	5717.5 (2752.5–56490.0)	0.002
Steroid duration (month) med (min-max)	84 (60–325)	34 (2–201)	<0.001
Antilipidemic n (%)	16 (7.5%)	0 (0%)	0.99
Antiaggregant n (%)	21 (9.8%)	1 (4.7%)	0.04
eGFR mean (±SD)	75.3 (±24.9)	68.8 (±23.6)	0.25
Ca, mmol/L mean (±SD)	2.38 (±0.15)	2.38 (±0.17)	0.92
P, mmol/l mean (±SD)	1.03 (±0.16)	1.00 (±0.19)	0.67
Mg, mmol/L mean (±SD)	0.85 (±0.15)	0.90 (±0.10)	0.78
25-OH-D, nmol/L mean (±SD)	79.6 (±28.7)	90.6 (±25.7)	0.71
Pre Tx iPTH, ng/L med (min-max)	573.5 (60.7–2854.2)	733.1 (141.2–2293.0)	0.06
1st year iPTH, ng/L med (min-max)	67.3 (17.5–2249.2)	53.2 (14.0–279.9)	0.16
Hb, g/L mean (±SD)	127.2 (±21.2)	120.1 (±16.1)	0.03
Alb, g/L mean (±SD)	43.2 (±4.0)	42.4 (±3.2)	0.72
LDL, mmol/L mean (±SD)	2.73 (±0.89)	2.54 (±0.66)	0.13
TG, mmol/L mean (±SD)	1.64 (±0.91)	1.55 (±0.74)	0.60
Follow-up (year) med (min-max)	7.0 (5.0–27.1)	9.0 (5.0–23.1)	0.14
Cdx2 CC/CT/TT n (%)	60.6/31.9/7.5	75.3/19.2/5.5	0.04
Fok1 AA/AG/GG n (%)	9.9/41.8/48.3	0.0/50.0/50.0	0.12
Bsm1 CC/CT/TT n (%)	37.6/41.3/21.1	27.3/59.1/13.6	0.03
Apa1 CC/CA/AA n (%)	23.0/38.0/39.0	18.2/59.1/22.7	0.02
Taq1 AA/AG/GG n (%)	40.4/40.4/19.2	31.9/54.5/13.6	0.09

The *VDR* gene’s BsmI CT or TT genotypes were found to increase the AVN risk with an HR of 3.48 (min: 1.11 – max: 10.42, P = 0.03). Higher hemoglobin levels were found to decrease the AVN risk with a hazard ratio (HR) of 0.76 (min: 0.58 – max: 0.99, P = 0.05), as shown in Table 8.

**Table 8:** Cox regression analysis of risk factors for avascular necrosis.

		HR (95% CI)	P value
Cdx2 (ref: CC)	CT or TT	0.40 (0.13–1.17)	0.09
FokI (ref: AA)	AG or GG	0.99 (0.44–3.44)	0.84
BsmI (ref: CC)	CT or TT	3.48 (1.11–10.42)	0.03
ApaI (ref: CC)	CA or AA	0.73 (0.04–12.99)	0.83
TaqI (ref: AA)	AG or GG	0.52 (0.03–9.02)	0.65
Steroid (mg)		1.00 (0.99–1.00)	0.01
Vintage (month)		0.91 (0.74–1.14)	0.43
25-OH-D (nmol/L)		1.04 (1.01–1.08)	0.01
Hb (g/L)		0.76 (0.58–0.99)	0.05
LDL (mmol/L)		0.99 (0.97–1.01)	0.35

### 3.5. Genotype distributions according to age and cumulative glucocorticoid doses and compound heterozygosity of the population

The distribution of Cdx2, Fok1, Bsm1, Apa1, and Taq1 polymorphisms according to age and cumulative glucocorticoid doses are shown in Table 9. The distributions of polymorphisms were similar in the groups under and above 50 years of age and among the groups receiving treatment below and above the mean cumulative glucocorticoid dose of the study population. 

**Table 9:** VDR polymorphism distributions according to age and cumulative steroid dose.

	Age <50	Age ≥50	P value	Lower cumulative steroid*	Higher cumulative steroid*	P value
Cdx2 CC/CT/TT (%)	103(58.9%) 56(32%) 16(9.1%)	39 (66.2%) 16 (27.1%) 4 (6.7%)	0.06	47 (55.3%) 32 (37.6%) 6 (7.1%)	98 (65.8%) 40 (26.8%) 11 (7.4%)	0.52
Fok1 AA/AG/GG (%)	18 (10.3%) 70 (40%) 87 (49.7%)	3 (5.1%) 30 (50.8%) 26 (44.1%)	0.12	7 (8.2%) 35 (41.2%) 43 (50.6%)	14 (9.4%) 65 (43.6%) 70 (47%)	0.93
Bsm1 CC/CT/TT (%)	64 (36.6%) 74 (42.3%) 37 (21.1%)	21 (35.6%) 27 (45.8%) 11 (18.6%)	0.66	36 (42.4%) 30 (35.3%) 19 (22.3%)	49 (32.9%) 71 (47.7%) 29 (19.4%)	0.07
Apa1 CC/CA/AA (%)	36 (20.6%) 72 (41.1%) 67 (38.3%)	16 (27.1%) 22 (37.3%) 21 (35.6%)	0.73	24 (28.2%) 30 (35.3%) 31 (36.5%)	28 (18.8%) 64 (43%) 57 (38.2%)	0.34
Taq1 AA/AG/GG (%)	68 (38.9%) 74 (42.3%) 33 (18.8%)	24 (40.7%) 24 (40.7%) 11 (18.6%)	0.89	39 (45.9%) 28 (32.9%) 18 (21.2%)	53 (35.6%) 70 (47%) 26 (17.4%)	0.06

Table 10 shows compound heterozygosity percentages of the study population. 70.8% of the cases with Cdx2 heterozygous polymorphism also carried a mutant ApaI polymorphism. All cases with BsmI heterozygous polymorphism also carried an additional mutation.

**Table 10:** VDR gene compound heterozygosity of the study population.

Cdx2 (CT/TT)		Additional mutations (%)				
		FokI(AG/GG)	BsmI(CT/TT)	ApaI(CA/AA)	TaqI(AG/GG)	
Heterozygous cases	Cdx2 CT (n = 72)	-	90.3%	58.3%	70.8%	58.3%
	FokI AG (n = 100)	35%	-	66%	75%	61%
	BsmI CT (n = 101)	37.6%	93%	-	100%	100%
	ApaI CA (n = 94)	36.1%	92.5%	69.1%	-	68%
	TaqI AG (n = 98)	37.7%	91.8%	97.9%	100%	-

## 4. Discussion

The current immunosuppression protocols after kidney transplantation provide a better graft survival but cause many complications that prominently impact bone health. The possibility of patients developing bone diseases with similar demographics, clinical features, or treatment protocols cannot be estimated. This is despite the significant factors of dialysis vintage, cumulative glucocorticoid dose, and age. This study investigated the essential *VDR* gene polymorphisms in a large kidney transplant recipient population and their significance within five or more years of follow-up.

The VDR is a 427-amino acid protein. The *VDR* gene is located on the long arm of chromosome 12 at position 13.11 (12q13.11). The gene consists of 11 exons [14]. The Cdx2 promoter polymorphism plays a role in the *VDR* gene’s intestinal-specific transcription. The G allele reduces *VDR* transcriptional activity relative to the A allele influencing its calcium absorption. The FokI polymorphism is located in the *VDR* gene exon 2 and plays an essential role in the VDR protein’s posttranscriptional modifications. The BsmI, ApaI, and TaqI polymorphisms are located in the 3’-regulatory region and are suggested to impact mRNA stability [15]. 

Glucocorticoids are the critical agents in bone mineral loss. The majority of mineral bone loss secondary to glucocorticoids occurs within the first year of treatment. Even with high BMD scores, this bone loss poses a greater bone fracture risk than postmenopausal osteoporosis [16]. In our study, the main differences between the normal and abnormal BMD groups with osteopenia or osteoporosis were the age, frequency of calcineurin inhibitor use, and first-year posttransplantation iPTH levels. The patients with abnormal BMD scores were significantly younger than the age mean. The reason for an abnormal BMD may be that younger patients receive more intense and prolonged glucocorticoid treatment and CNIs when their primary kidney disease diagnosis is glomerulonephritis or vasculitis. It is unknown if patients who underwent these therapies for their primary kidney disease also accessed such bone-sparing measures as vitamin D-calcium supplementation and DEXA monitoring. The subjects using CsA were a majority in the abnormal BMD group. The previous studies demonstrated that the CsA alone or with steroids increases a patient’s osteoporosis risk [5, 17–19]. 

It was not pretransplantation, but the first-year posttransplantation iPTH levels were significantly higher in the abnormal BMD group. With the exception of age, the differences between the groups lost their significance in the regression analysis for an abnormal BMD risk. The *VDR* gene’s homozygous Cdx2 polymorphism was associated with a 3.34 fold increase in the abnormal first-year BMD scores’ risk. The high serum phosphorus levels in the first-year posttransplantation were significantly protective of the low BMD scores.

The relationship between the *VDR* gene polymorphisms and osteoporosis was mainly studied in postmenopausal women. FokI, BsmI, and ApaI polymorphisms are associated with postmenopausal osteoporosis in several studies, but metaanalyses contradict these studies [20, 21]. The predisposition to postmenopausal osteoporosis caused by TaqI polymorphism is controversial. The potential origin of such controversy is because TaqI polymorphism is a silent polymorphism. It does not cause an amino acid change in contrast to BsmI and ApaI polymorphisms [15]. No comprehensive studies exist with Cdx2 polymorphism. More importantly, studies on kidney transplant recipients are limited. Postmenopausal osteoporosis has a diverse population and conditions. Lower BMD results were observed in the younger renal transplant recipients in the study of Özel et al. [6]. Although the age factor lost its significance in the regression analysis, similar findings were found in our study. In the same study, BsmI polymorphisms were frequent in patients with osteoporosis after kidney transplantation, but a regression analysis was not applied. In our study, BsmI was not a significant risk factor in the regression analysis when corrected with essential clinical factors for osteoporosis.

When the subjects were evaluated in hyperparathyroidism at the end of the first year of transplantation, the persistent hyperparathyroidism group had a longer dialysis vintage. We found the pretransplantation iPTH levels to be significantly higher. In the regression analysis, these findings lost their significance. The risk of first-year abnormal BMD scores was increased 6.81 times by heterozygous Cdx2 polymorphism, 23.32 times by homozygous Cdx2 polymorphism, 4.01 times by heterozygous ApaI polymorphism, and 6.30 times by homozygous ApaI polymorphism. Several studies investigated the *VDR* polymorphisms with hyperparathyroidism secondary to CKD. The study results were contradictory when examining the relationship between BsmI polymorphism and secondary hyperparathyroidism [8, 22–25]. FokI polymorphism was associated with higher iPTH values in dialysis patients [26].

The most unpredictable of all posttransplant bone complications is AVN. The leading risk factor for AVN is the use of glucocorticoids. However, the predictive value of the treatment duration and cumulative doses of glucocorticoids are controversial [27–29]. The studies investigating an AVN susceptibility have generally included coagulation cascade-associated genes [30]. Two studies investigated the risk of FokI polymorphisms on AVN development in pediatric leukemia patients but found conflicting results with each other [31]. When our study population with a minimum follow-up period of five years was evaluated, the cumulative dose of glucocorticoid treatment was significantly lower, and the duration of glucocorticoid use was significantly shorter in patients who developed AVN. When corrected by all proposed risk factors, the presence of BsmI heterozygous or a homozygous polymorphism of the *VDR* gene increased the risk of AVN by 3.48 times. Also, the higher hemoglobin levels were found to be protective against AVN development. The relationship between hemoglobin and AVN was mainly investigated in patients with sickle cell disease, and an association between anemia and AVN was shown [32]. Narayanan et al. reported significantly lower hemoglobin levels in nonsickle cell disease patients with AVN. These results are consistent with the findings in our study [33]. In this respect, the higher hemoglobin levels may increase the bone’s oxygenation and demonstrate a protective effect. Anemia control of kidney transplantation recipients may be crucial for protection from AVN. However, the regression analysis revealed that the glucocorticoid treatment’s cumulative dose or duration was not associated with an AVN risk. 

Other observations in this study are that although not as severe as the mutant types, heterozygous variants of Cdx2 and ApaI polymorphisms and the heterozygous variant of BsmI is also associated with an increased risk of persistent secondary hyperparathyroidism and AVN; respectively. Compound heterozygosity is the condition of having two or more heterozygous or mutant alleles at a particular locus that can cause genetic disease in a heterozygous state [34]. We observed that the coexistence of heterozygous *VDR* gene mutations in our study population is relatively high. An ApaI mutation was detected in 70.8% of Cdx2 heterozygous cases, and an additional mutation was detected in all of the BsmI heterozygous cases. In this context, it is impossible to say whether the BsmI mutation itself is a risk factor or poses a risk due to accompanying mutations. Studies with larger populations are necessary to elucidate the risks of compound heterozygosity in this patient population.

The study’s limitation is that although the number of patients is large, it is single centered. Multicenter and multinational studies will determine the subject’s availability in clinical practice. If supported by subsequent studies, the immunosuppressive agents’ management after transplantation and bone health measures can be individualized.

## 5. Conclusion

Bone diseases are one of the significant morbidities after kidney transplantation. The bone diseases that affect kidney transplant recipients are osteoporosis and AVN. Both osteoporosis and AVN cause significant, long-term morbidity. Kidney transplant recipients are also indefensible to persistent hyperparathyroidism because of their longstanding history of CKD. This increases the complexity of the evaluation and the treatment of mineral and bone disorders. This study suggests that *VDR* gene polymorphisms are relevant in patients’ bone health after kidney transplantation. If these polymorphisms are studied in a more extensive series, and similar findings are detected, they will help identify high-risk patients. Necessary measures can be taken for these patients by focusing on and tailoring their therapy accordingly.

## Informed consent

The study protocol was approved by the Ethical Committee on Human Research of Gazi University Faculty of Medicine with the approval number 34 on January 22th, 2018. All patients participating in the study provided informed consent.
